# Integrative effects of different mulching practices and *Azospirillum brasilense* on wheat growth, physiology, and soil health under drought stress

**DOI:** 10.1038/s41598-025-19031-5

**Published:** 2025-09-29

**Authors:** Kamran Ikram, Muhammad Zeeshan Mansha, Khalid Mahmood, Akhtar Hameed, Muhammad Mubashir Omar, Rana Mubashar Hassan, Luqman Amrao, Muhammad Saqlain Zaheer, Noman Ali Buttar, Yasir Niaz, Nadeem Ahmed, Rubab Iqbal, Siqi Lu, Muhammad Rizwan, Khairiah Mubarak Alwutayd

**Affiliations:** 1 Department of Agricultural Engineering, Khwaja Fareed University of Engineering and Information Technology, Rahim Yar Khan, Pakistan; 2 Department of Plant Pathology, University of Layyah, Layyah, Pakistan; 3 Department of Chemical & Polymer Engineering, University of Engineering & Technology Lahore, Faisalabad Campus, 3½ Km. Khurrianwala - Makkuana By-Pass, Faisalabad, Pakistan; 4 Institute of Plant Protection, MNS University of Agriculture, Multan, 61000 Pakistan; 5 Department of Energy Systems Engineering, University of Agriculture Faisalabad, Faisalabad, 38000 Pakistan; 6 Department of Agriculture, Government of Punjab, Punjab, Pakistan; 7 Department of Plant Pathology, University of Agriculture, Faisalabad, Faisalabad, 38000 Pakistan; 8 Fundación CEAM, c/ Charles R. Darwin 14, Parque Tecnológico, Paterna, Valencia Spain; 9 Department of Botany, University of Agriculture Faisalabad, Faisalabad, 3800 Pakistan; 10 Department of Geography, Sustainability, Community, and Urban Studies, University of Connecticut, Storrs, CT 06269-4148 USA; 11 Institute of Crop Science and Resource Conservation (INRES), University of Bonn, 53115 Bonn, Germany; 12 Department of Biology, College of Science, Princess Nourah Bint Abdulrahman University, P.O. Box 84428, 11671 Riyadh, Saudi Arabia

**Keywords:** Drought stress, Soil microbes, Crop physiology, Soil nutrients, Wheat growth, Ecology, Ecology, Environmental sciences, Physiology, Plant sciences

## Abstract

*Azospirillum brasilense*, a plant growth-promoting rhizobacterium that plays a vital role in sustainable wheat production by enhancing nutrient uptake, improving stress tolerance, and reducing reliance on chemical fertilizers. This study aimed to investigate the integrative effects of *Azospirillum brasilense* inoculation and different mulching practices on the growth, physiology, and soil health of wheat (*Triticum aestivum* L.) under drought stress, particularly during the critical booting stage. The primary research question focused on identifying whether these combined treatments could mitigate drought-induced damage and enhance plant performance. The experimental was consisted of 9 treatments, including T0 (control: no mulch, no drought, no soil microbes), T1 (drought stress at the booting stage (DB)), T2 (DB + *A. brasilense*), T3 (DB + wheat straw mulch), T4 (DB + rice husk mulch), T5 (DB + plastic mulch), T6 (DB + *A. brasilense* + wheat straw mulch), T7 (DB + *A. brasilense* + rice husk mulch), and T8 (DB + *A. brasilense* + plastic mulch) with randomized complete block design having three replications. Various growth, yield, physiological, and soil nutrient parameters were assessed. Data analysis included ANOVA, cluster heatmap, and principal component analysis (PCA) to evaluate treatment impacts. Drought stress significantly reduced plant height (34.24%), 1000-grain weight (49.05%), and photosynthetic pigments. However, treatments combining *A. brasilense* with organic mulches (T6: wheat straw and T7: rice husk) substantially improved plant biomass, photosynthetic rate (up to 24.67%), stomatal conductance (7.54%), and soil nutrient uptake. T6 showed the highest increase in chlorophyll a (118.74%) and grain weight (78.78%) compared to drought alone. PCA and heatmap analyses revealed strong clustering of treatments, highlighting T6 as the most effective strategy. The combination of *A. brasilense* and organic mulching (especially wheat straw) effectively mitigated drought stress in wheat by enhancing physiological resilience, nutrient uptake, and soil health. The demonstrated benefits suggest that incorporating bio-inoculants with locally available mulching materials can be scaled up as a practical intervention for climate-smart agriculture.

## Introduction

Wheat is a staple food in Pakistan. Approximately 36% of the world’s population consumes wheat as a stable food^1^. Compared with other cereals, wheat grains constitute the greatest percentage of food calories (20%) and carbohydrates (36%), in addition to various minerals, antioxidants and vitamins^2,3^. The area of cultivated wheat is increasing gradually around the globe, with increasing demand for wheat. It can be grown under diverse climate conditions^4,5^. If the population continues to increase at the current growth rate, 60% of the current food production needs to be increased by the middle of the century to meet food security requirements^6^. However, the current pace of wheat production is not sufficient to meet the increasing global food demand^7^.

Water is essential for survival. Water deficiency affects plant growth and overall crops in terms of poor grain quality and low production per unit area^8^. In recent decades, the complex phenomenon of climate change has created challenges for researchers and producers in meeting increasing food demand^9,10^. Drought is one of the main effects of climate change on crop production^11,12^. Other factors, such as salinity and heavy metal accumulation in plant tissues, also influence crop yield^4,13^. In drought situations, water is not available to plants for a prolonged period of time^14^. A total of 34.9% of the world’s area is under arid and semiarid climate conditions, of which less than 15% has irrigation facilities. The rest of the area is rain-fed^14^. In the past, due to drought and heat waves, global wheat production was reduced by up to 35%^8^. This creates an alarming situation in which more food production is needed to feed the increasing population.

Plant species respond to drought in either a deleterious or adaptive way^15^. Plants with deleterious responses exhibit poor growth, low water-use efficiency, lost turgor, low photosynthesis and transpiration, damaged macromolecules and cell membranes, altered enzyme activities and metabolism, greater reactive oxygen species production and reduced yields^16,17^. Some plants have the ability of natural drought tolerance. Drought tolerance is the ability of plants to survive and achieve significant production under periodic drought conditions^18^. To mitigate drought conditions, these plants change their physiology, root structure and production^18,19^. These plants maintain water uptake by promoting root development, reducing water loss through closing stomata, limiting the shoot growth rate, activating antioxidative defense systems, stabilizing the cell membrane and regulating hormone activity for survival under prolonged drought conditions^16,20^.

Plant growth-promoting bacteria are beneficial for plant physiological processes under both normal and drought conditions^21^. These bacteria can also increase the photosynthesis rate and nutrient uptake in plants^22^. The genus *Azospirillum* comprises free-living and nitrogen-fixing bacteria that can promote plant growth. The application of *A. brasilense* is considered a viable economic and eco-friendly technology^23^. These bacteria promote crop growth under normal and water-stressed conditions^21,24,25^. These bacteria promote nitrogen fixation and plant growth^26^, significantly contribute to plant‒soil nitrogen management^27^, and are beneficial for increasing the zinc concentration in seeds^28,29^. These bacteria also improve plant drought tolerance through direct or indirect mechanisms^21^. They improve a plant’s antioxidant defense system^24^, proline accumulation^30^ and abscisic acid levels^31^. Many mulching techniques, including straw mulch, plastic mulch and ridge-furrow mulch, have been proven to be beneficial. Plastic mulch remains in the field and has negative effects on the environment and soil in terms of water and nutrient transport, resulting in low crop yields^32^. Straw mulching is also a widely adopted technique in arid and semiarid climates where drought is a severe issue because of its ability to retain moisture and hence improve crop production^33,34^. It maintains moisture in the soil by reducing evaporation losses, reducing soil heat loss to the air and improving the water retention ability of the soil^35^. Straw mulch provides significant amounts of potassium, nitrogen, phosphorous and other essential nutrients and improves the soil quality of the next crop^36^. A literature review revealed the combined effects of straw mulch and *A. brasilense* bacteria on the physiology and yield of wheat crops under drought conditions. Given the water deficit conditions and importance of wheat crops, a study was designed to investigate the combined effects of wheat straw mulch and *A. brasilense* on wheat plant physiology and crop yield. We hypothesized that the combined application of *A. brasilense* and mulching practices would more effectively alleviate drought-induced stress in wheat than either treatment alone. Specifically, we anticipated that organic mulches (wheat straw and rice husk), by conserving soil moisture and enhancing nutrient availability, would complement the physiological and soil health benefits provided by *A. brasilense*. Therefore, the purpose of this study was to evaluate the integrative effects of different mulching practices and *A. brasilense* inoculation on wheat growth, physiology, and soil health under drought stress.

## Materials and methods

### Growth conditions and experimental layout

**Fig. 1:**
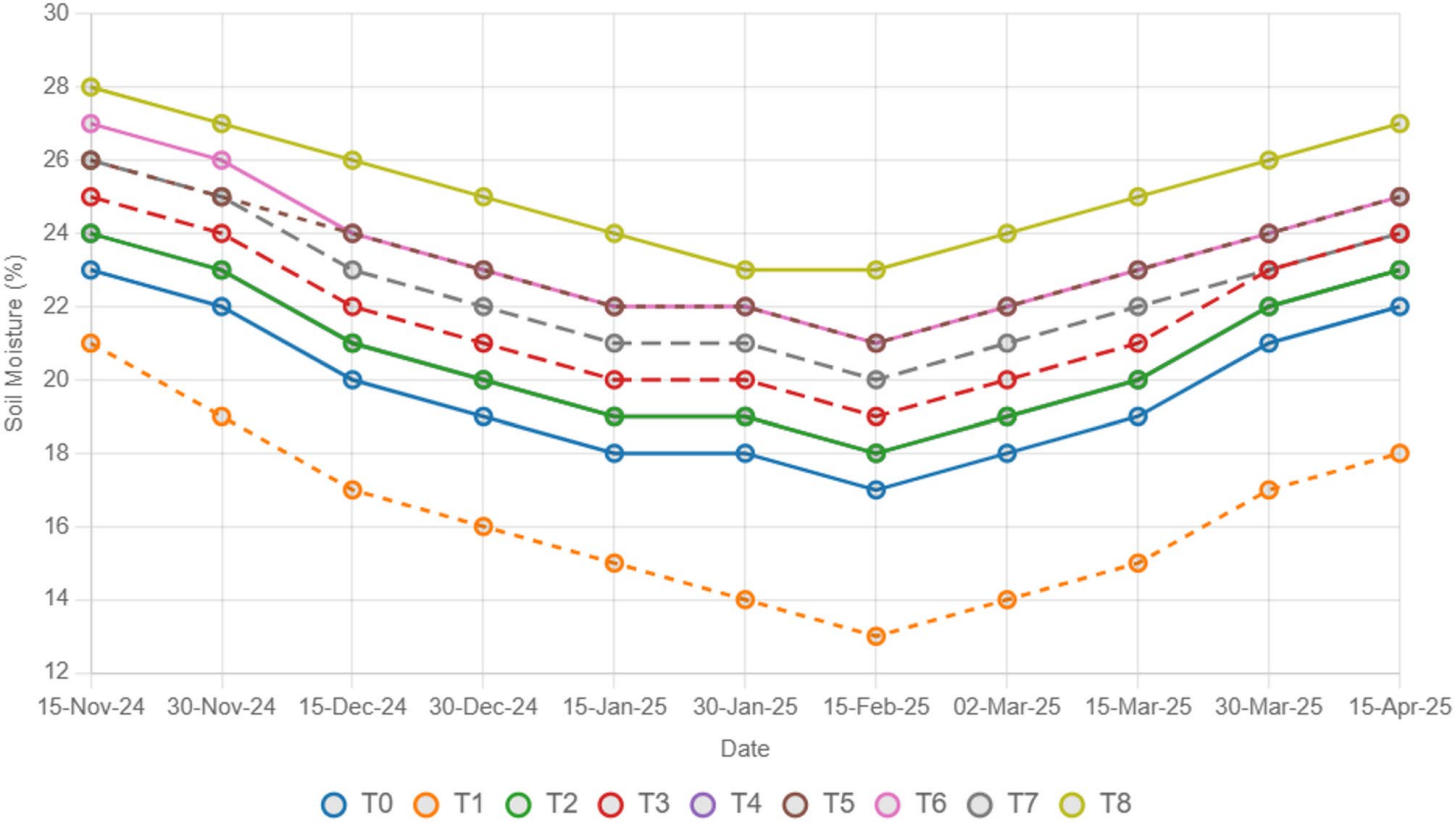
Effect of different treatments on soil moisture (%) of the experimental soil.

**Fig. 2:**
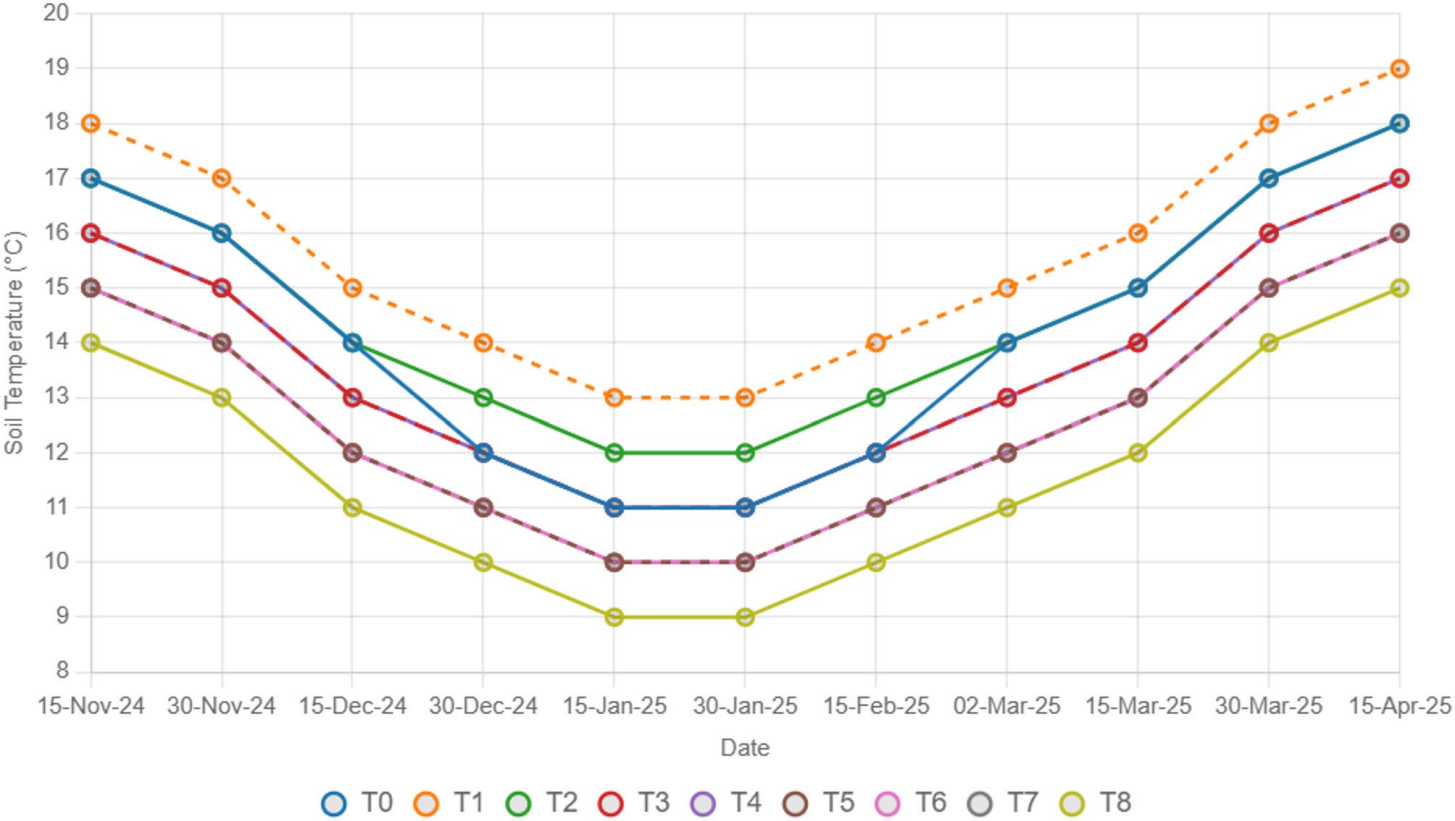
Effect of different treatments on soil temperature (°C) of the experimental soil.

To estimate the combined effects of different types of straw mulch and *A. brasilense* on wheat crops, this study was conducted at the agricultural research farms of the department of agricultural engineering, khwaja fareed university of engineering and information technology, Rahim Yar Khan, Pakistan (Latitude ^28°^ 25’ 3’’ N, Longitude 70° 18’ 13’’ E). The Cholistan desert surrounds the study area. The weather is hot and dry, with an average temperature of 26.5 °C. The average precipitation in the study area is 101 mm^37^. The study was designed under a randomized complete block design (RCBD) with three replications. Wheat seeds of an approved wheat variety (Ujala-16) were obtained from the regional agriculture research institute, Bahawalpur, Pakistan, and *A. brasilense* was obtained from the ayub agriculture research center, Faisalabad, Pakistan. The fields were prepared via standard tillage practices, and seeds were sown via a standard wheat drill. The seed drill was calibrated for a seed rate of 50 kg/acre. The plot size for each treatment was 5 × 7 feet. The recommended NPK fertilizer dose was applied in the field, and an accurate amount for each plot was calculated as 120:80:60 NPK kg/ha^1^. Sowing of the wheat seeds was done on 15th November 2024 and the harvesting date was 25th April, 2025. All treatments, including control and stress treatments, received the same recommended fertilizer dose to ensure uniform nutrient availability. Three replications for each treatment were selected, and 27 plots were prepared. The experimental setup consisted of 9 treatments, including T_0_ (control: no mulch, no drought, no soil microbes), T_1_ (drought stress at the booting stage (DB)), T_2_ (DB + *A. brasilense*), T_3_ (DB + wheat straw mulch), T_4_ (DB + rice husk mulch), T_5_ (DB + plastic mulch), T_6_ (DB + *A. brasilense* + wheat straw mulch), T_7_ (DB + *A. brasilense* + rice husk mulch), and T_8_ (DB + *A. brasilense* + plastic mulch). For the mulching treatments, a 2-inch thick layer of wheat straw or rice straw was applied uniformly across the field, and a plastic sheet was spread over the field to reduce evaporation losses and measure its effects on crop production. Drought stress was applied after 50 days of sowing at the booting stage, withholding irrigation water, and data were collected as described below. Soil moisture (Fig. 1) and temperature (Fig. 2) are measured at 0–15 cm during the whole crop period with the help of a soil moisture sensor and digital soil thermometer sensor.

Recorded parameters: Plant growth parameters, such as plant height, root length, shoot length, spike length, number of spikelets, leaf area index, ear length, 1000-grain weight and grain weight per plant, were recorded at the time of crop harvesting via standard procedures^3,38,39^. The crop growth rate (CGR) was calculated via the standard procedure^40^ using the following formula,

$${\text{CGR }} = {\text{ }}\left\{ {\left( {{\text{W2 }} - {\text{ W1}}} \right){\text{ }}/{\text{ }}\left( {{\text{t2}} - {\text{t1}}} \right)} \right\}{\text{ }} \times {\text{ 1}}/{\text{A}}$$

Where W1 is the dry weight of the crop at the time of t1 and W2 is the dry weight of the crop at time t2. T2-t1 is the time interval, and A is the ground area occupied by the crop.

The NPK in plant shoots was determined via the standard procedure reported in previous research^41^. The MDA content and electrolyte leakage were measured at the anthesis stage by harvesting fresh leaves. The MDA content in wheat leaves is used as an indicator of drought-induced lipid peroxidation. The MDA in wheat leaves was measured following the standard procedure^42^, whereas electrolyte leakage was measured via the standard method of a previous research^43^. Free proline contents, photosynthesis rate and carotenoids were measured via the standard techniques described in a an earlier research^44,45^. We also follow the previous researchers for chlorophylls^46^ and leaf area calculations^16^. The photosynthetic rate was measured via a gas analyzer, stomatal conductance was recorded at the anthesis stage, and stomatal conductance was measured via the standard procedure described by a previous researcher^1^. All physiological and growth related parameters were recorded at the grain filling stage and yield related parameters were recorded after the crop harvesting.

### Statistical analysis

An analysis of variance (ANOVA) was conducted on the dataset to explore potential statistically significant differences and dominant patterns among the applied treatments. Pearson’s correlation analysis was used to investigate the linkages and associations between the variables. The data were analyzed via the statistical software package Statistix v. 8.01. The statistical and visualization tool of the R-Studio program was used to conduct principal component analysis, heatmaps and correlation analysis. Heatmap was generated using the heatmap package (v5.5.1) in R “v4.5.1”, https://cran.r-project.org/bin/windows/base/.

## Results

### Plant growth and yield parameters

**Fig. 3:**
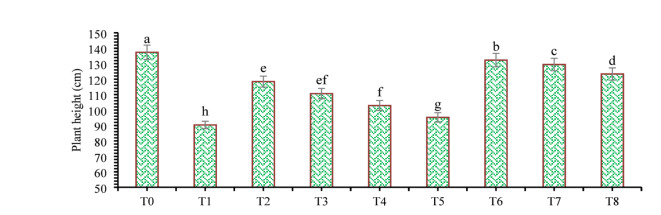
Effects of different mulching practices and *Azospirillum brasilense* on the plant height of Wheat under drought. T_0_ (control: no mulch, no drought, no soil microbes), T_1_ (drought stress at the booting stage (DB)), T_2_ (DB + *A. brasilense*), T_3_ (DB + wheat straw mulch), T_4_ (DB + rice husk mulch), T_5_ (DB + plastic mulch), T_6_ (DB + *A. brasilense* + wheat straw mulch), T_7_ (DB + *A. brasilense* + rice husk mulch), and T_8_ (DB + *A. brasilense* + plastic mulch). Bars marked with different letters indicate significant differences between treatments at *p* < 0.05.

**Fig. 4:**
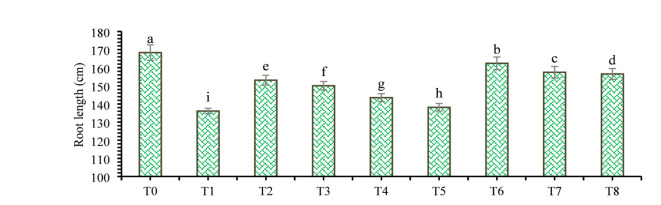
Effects of different mulching practices and *Azospirillum brasilense* on the root length of wheat under drought. T_0_ (control: no mulch, no drought, no soil microbes), T_1_ (drought stress at the booting stage (DB)), T_2_ (DB + *A. brasilense*), T_3_ (DB + wheat straw mulch), T_4_ (DB + rice husk mulch), T_5_ (DB + plastic mulch), T_6_ (DB + *A. brasilense* + wheat straw mulch), T_7_ (DB + *A. brasilense* + rice husk mulch), and T_8_ (DB + *A. brasilense* + plastic mulch). Bars marked with different letters indicate significant differences between treatments at *p* < 0.05.

**Fig. 5:**
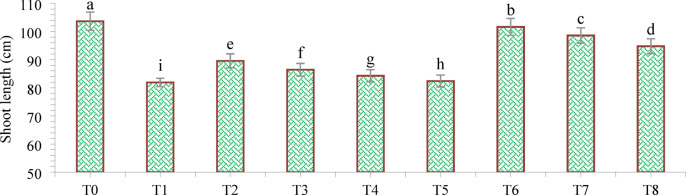
Effects of different mulching practices and *Azospirillum brasilense* on the shoot length of Wheat under drought. T_0_ (control: no mulch, no drought, no soil microbes), T_1_ (drought stress at the booting stage (DB)), T_2_ (DB + *A. brasilense*), T_3_ (DB + wheat straw mulch), T_4_ (DB + rice husk mulch), T_5_ (DB + plastic mulch), T_6_ (DB + *A. brasilense* + wheat straw mulch), T_7_ (DB + *A. brasilense* + rice husk mulch), and T_8_ (DB + *A. brasilense* + plastic mulch). Bars marked with different letters indicate significant differences between treatments at *p* < 0.05.

**Fig. 6:**
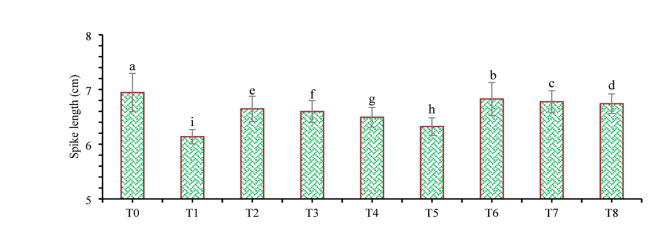
Effects of different mulching practices and *Azospirillum brasilense* on the spike length of wheat under drought. T_0_ (control: no mulch, no drought, no soil microbes), T_1_ (drought stress at the booting stage (DB)), T_2_ (DB + *A. brasilense*), T_3_ (DB + wheat straw mulch), T_4_ (DB + rice husk mulch), T_5_ (DB + plastic mulch), T_6_ (DB + *A. brasilense* + wheat straw mulch), T_7_ (DB + *A. brasilense* + rice husk mulch), and T_8_ (DB + *A. brasilense* + plastic mulch). Bars marked with different letters indicate significant differences between treatments at *p* < 0.05.

**Fig. 7:**
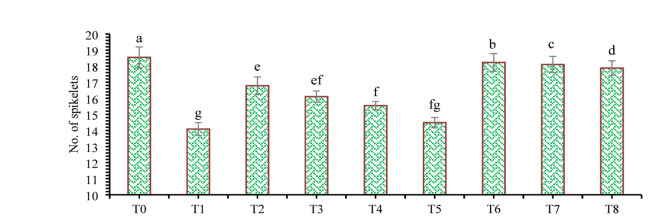
Effects of different mulching practices and *Azospirillum brasilense* on the no. of spikelets of wheat under drought. T_0_ (control: no mulch, no drought, no soil microbes), T_1_ (drought stress at the booting stage (DB)), T_2_ (DB + *A. brasilense*), T_3_ (DB + wheat straw mulch), T_4_ (DB + rice husk mulch), T_5_ (DB + plastic mulch), T_6_ (DB + *A. brasilense* + wheat straw mulch), T_7_ (DB + *A. brasilense* + rice husk mulch), and T_8_ (DB + *A. brasilense* + plastic mulch). Bars marked with different letters indicate significant differences between treatments at *p* < 0.05.

**Fig. 8:**
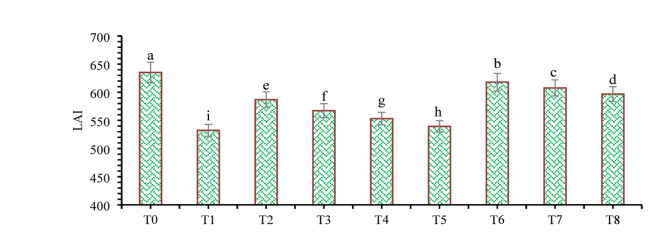
Effects of different mulching practices and *Azospirillum brasilense* on the leaf area index (LAI) of wheat under drought. T_0_ (control: no mulch, no drought, no soil microbes), T_1_ (drought stress at the booting stage (DB)), T_2_ (DB + *A. brasilense*), T_3_ (DB + wheat straw mulch), T_4_ (DB + rice husk mulch), T_5_ (DB + plastic mulch), T_6_ (DB + *A. brasilense* + wheat straw mulch), T_7_ (DB + *A. brasilense* + rice husk mulch), and T_8_ (DB +* A. brasilense* + plastic mulch). Bars marked with different letters indicate significant differences between treatments at *p* < 0.05.

**Fig. 9:**
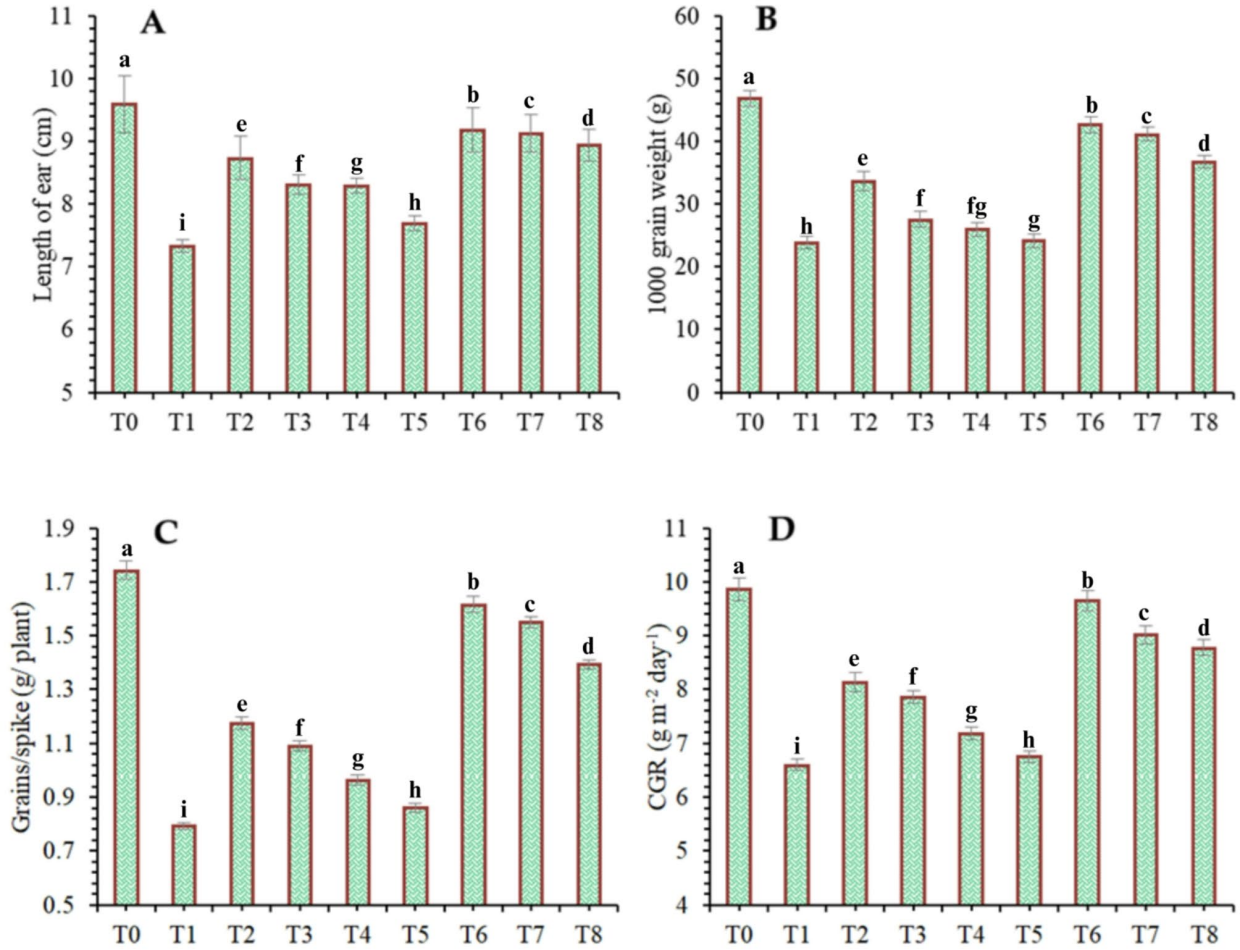
Effects of different mulching practices and *Azospirillum brasilense* on the length of ear, 1000-grain weight, grain per spike and crop growth rate (CGR) of Wheat under drought. T_0_ (control: no mulch, no drought, no soil microbes), T_1_ (drought stress at the booting stage (DB)), T_2_ (DB + *A. brasilense*), T_3_ (DB + wheat straw mulch), T_4_ (DB + rice husk mulch), T_5_ (DB + plastic mulch), T_6_ (DB + *A. brasilense* + wheat straw mulch), T_7_ (DB + *A. brasilense* + rice husk mulch), and T_8_ (DB + *A. brasilense* + plastic mulch). Bars marked with different letters indicate significant differences between treatments at *p* < 0.05.

**Fig. 10:**
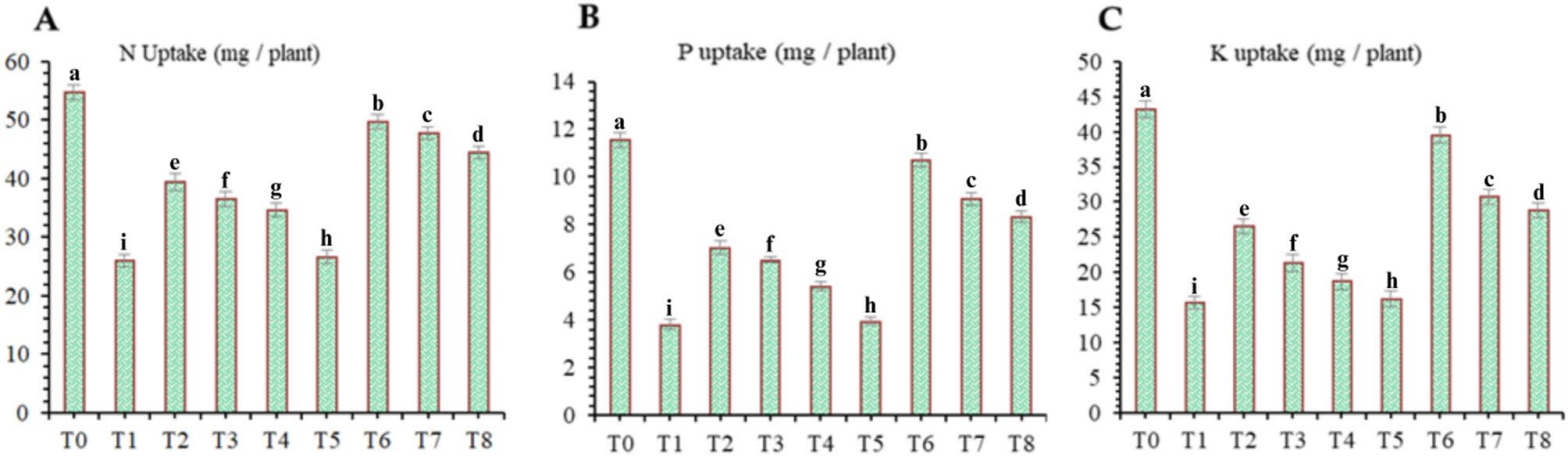
Effects of different mulching practices and *Azospirillum brasilense* on straw nitrogen (N), phousporus (P) and potassium (K) uptake wheat under drought. T_0_ (control: no mulch, no drought, no soil microbes), T_1_ (drought stress at the booting stage (DB)), T_2_ (DB + *A. brasilense*), T_3_ (DB + wheat straw mulch), T_4_ (DB + rice husk mulch), T_5_ (DB + plastic mulch), T_6_ (DB + *A. brasilense* + wheat straw mulch), T_7_ (DB + *A. brasilense* + rice husk mulch), and T_8_ (DB +* A.* *brasilense* + plastic mulch). Bars marked with different letters indicate significant differences between treatments at *p* < 0.05.

The findings of this study, which were based on data analysis, revealed that drought stress affected the growth and yield parameters of wheat plants under in vivo conditions. A linear decrease in all the growth and yield parameters was observed with increasing severity of drought stress. However, the addition of various treatments, viz., mulching and *A. brasilense*, significantly (*p* ≤ 0.05) improved the growth and yield characteristics of the wheat plants under drought-stressed conditions (Fig. 3). The positive control (T_0_) presented a greater ratio of growth and yield parameters than did the other treatments. Compared with normal (positive control) conditions, drought stress (negative control, T_1_) decreased the plant height (34.24%) (Fig. 1), root length (19.15%) (Fig. 4), shoot length (21.01%) (Fig. 5), spike length (11.62%) (Fig. 6), number of spikelets (24.05%) (Fig. 7), leaf area index (16.18%) (Fig. 8), ear length (23.66%), 1000-grain weight (49.05%), spike per plant (54.49%), CGR (33.06%) (Fig. 9), straw N uptake (52.57%), straw P uptake (67.07%) and straw K uptake (63.76%) (Fig. 10). Compared with the negative control (T1), the combined application of DB + *A. brasilense* + wheat straw mulch (T_6_) increased the plant height (46.45%), root length (19.32%), shoot length (24.09%), spike length (11.24%), number of spikelets (29.41%), leaf area index (16.06%), ear length (25.30%), 1000-grain weight (78.78%), spike per plant (103.78%), CGR (46.21%), straw N uptake (91.17%), straw P uptake (181.43%) and straw K uptake (152.49%).

### Plant physiological parameters

#### Malondialdehyde (MDA), electrolyte leakage and proline contents

**Fig. 11:**
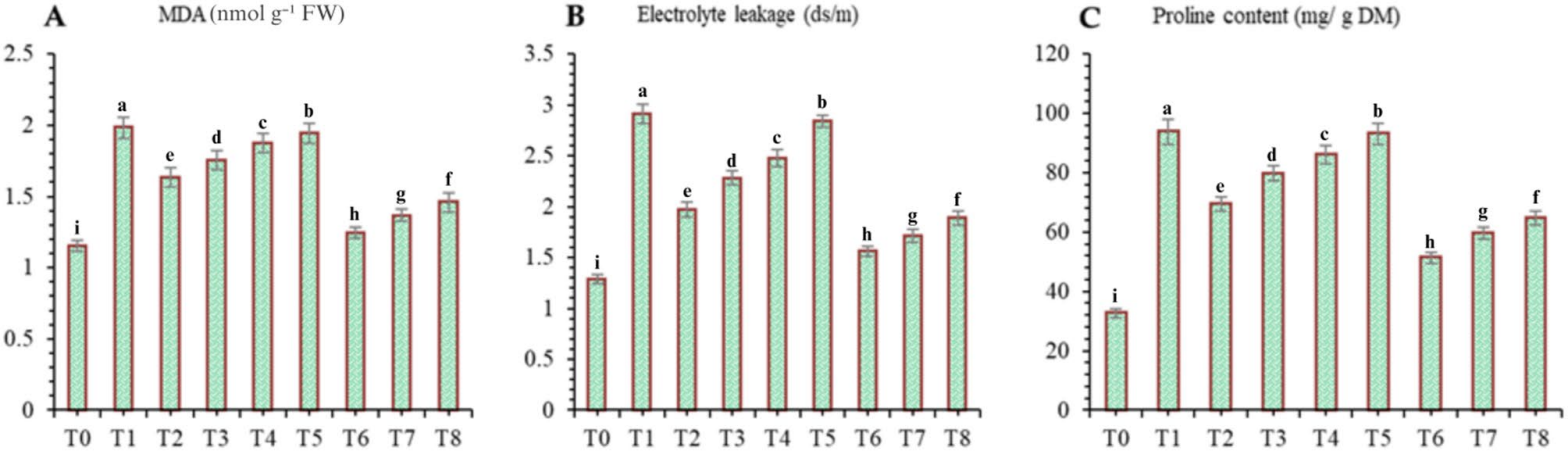
Effects of different mulching practices and *Azospirillum brasilense* on malondialdehyde (MDA) contents (**A**), electrolyte leakage (**B**) and proline content (**C**) of wheat under drought. T_0_ (control: no mulch, no drought, no soil microbes), T_1_ (drought stress at the booting stage (DB)), T_2_ (DB + *A. brasilense*), T_3_ (DB + wheat straw mulch), T_4_ (DB + rice husk mulch), T_5_ (DB + plastic mulch), T_6_ (DB + *A. brasilense* + wheat straw mulch), T_7_ (DB + *A. brasilense* + rice husk mulch), and T_8_ (DB + *A. brasilense* + plastic mulch). Bars marked with different letters indicate significant differences between treatments at *p* < 0.05.

Compared with the positive control (T0), the negative control (T1) resulted in a greater percentage of MDA (71.97%), electrolyte leakage (126.75%) and proline (187.15%) in wheat. However, the addition of DB + *A. brasilense* + wheat straw mulch (T_6_) significantly (*p* ≤ 0.05) impacted the measured parameters, as the contents of MDA (37.31%), electrolyte leakage (46.39%) and proline (45.26%) were lower than those of the negative control (T_1_) but greater than those of the positive control (T_0_) under drought conditions (Fig. 11).

#### Photosynthesis pigments, photosynthesis rate and stomatal conductance parameters

**Fig. 12:**
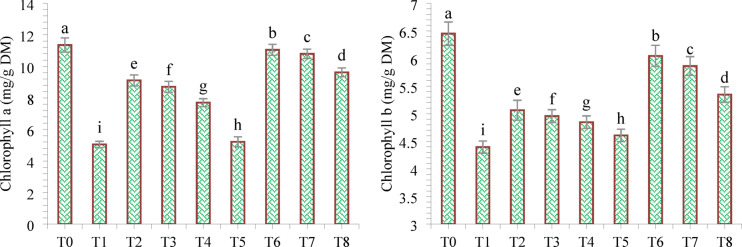
Effects of different mulching practices and *Azospirillum brasilense* on chlorophyll a and b of wheat under drought. T_0_ (control: no mulch, no drought, no soil microbes), T_1_ (drought stress at the booting stage (DB)), T_2_ (DB + *A. brasilense*), T_3_ (DB + wheat straw mulch), T_4_ (DB + rice husk mulch), T_5_ (DB + plastic mulch), T_6_ (DB + *A. brasilense* + wheat straw mulch), T_7_ (DB + *A. brasilense* + rice husk mulch), and T_8_ (DB +* A. brasilense* + plastic mulch). Bars marked with different letters indicate significant differences between treatments at *p* < 0.05.

**Fig. 13:**
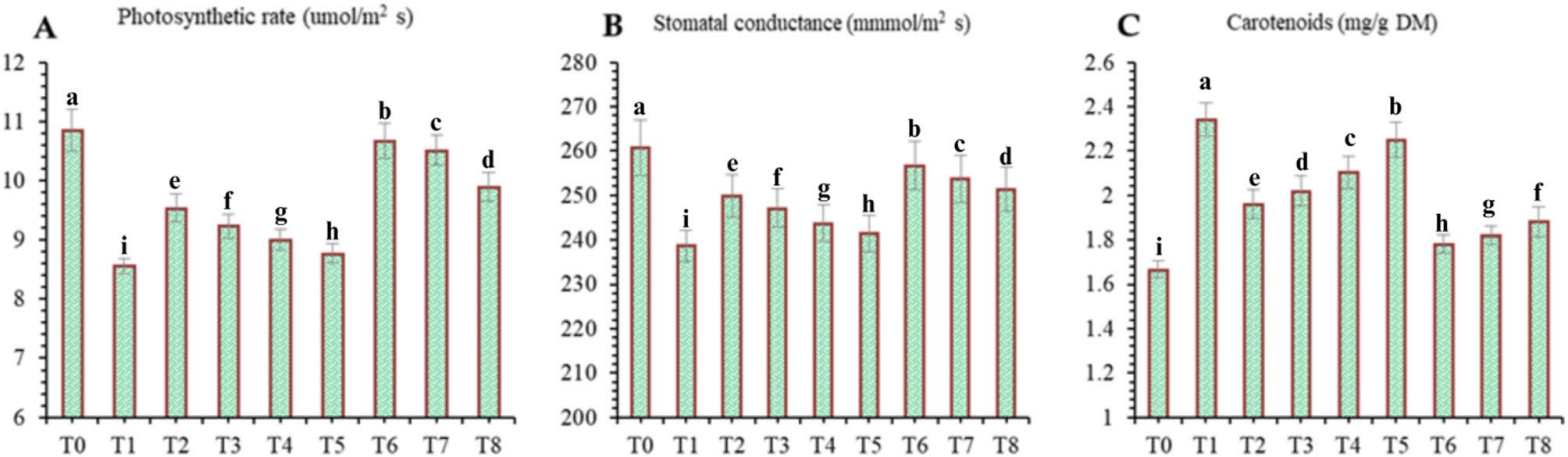
Effects of different mulching practices and *Azospirillum brasilense* on photosynthesis rate, stomatal conductance and carotenoids of Wheat under drought. T_0_ (control: no mulch, no drought, no soil microbes), T_1_ (drought stress at the booting stage (DB)), T_2_ (DB + *A. brasilense*), T_3_ (DB + wheat straw mulch), T_4_ (DB + rice husk mulch), T_5_ (DB + plastic mulch), T_6_ (DB + *A. brasilense* + wheat straw mulch), T_7_ (DB + *A. brasilense* + rice husk mulch), and T_8_ (DB + *A. brasilense* + plastic mulch). Bars marked with different letters indicate significant differences between treatments at *p* < 0.05.

Compared with the negative control treatment, various treatments significantly improved the synthesis of photosynthetic pigments in wheat under drought stress (Fig. 12). Compared with all the other treatments, the drought stress (T_1_) treatment resulted in the greatest decrease in the accumulation of photosynthetic attributes. However, the application of DB + *A. brasilense* + wheat straw mulch (T_6_) had the greatest effect on improving the chlorophyll content and stomatal conductance in wheat leaves. Compared with the negative control (T1), the DB + *A. brasilense* + wheat straw mulch (T_6_) treatment increased the chlorophyll a content (118.74%), chlorophyll b content (37.52%), photosynthetic rate (24.67%) and stomatal conductance (7.54%), In contrast, thecarotenoid content was greater in the negative control (T1) (Fig. 13).

### Correlation matrix

**Fig. 14:**
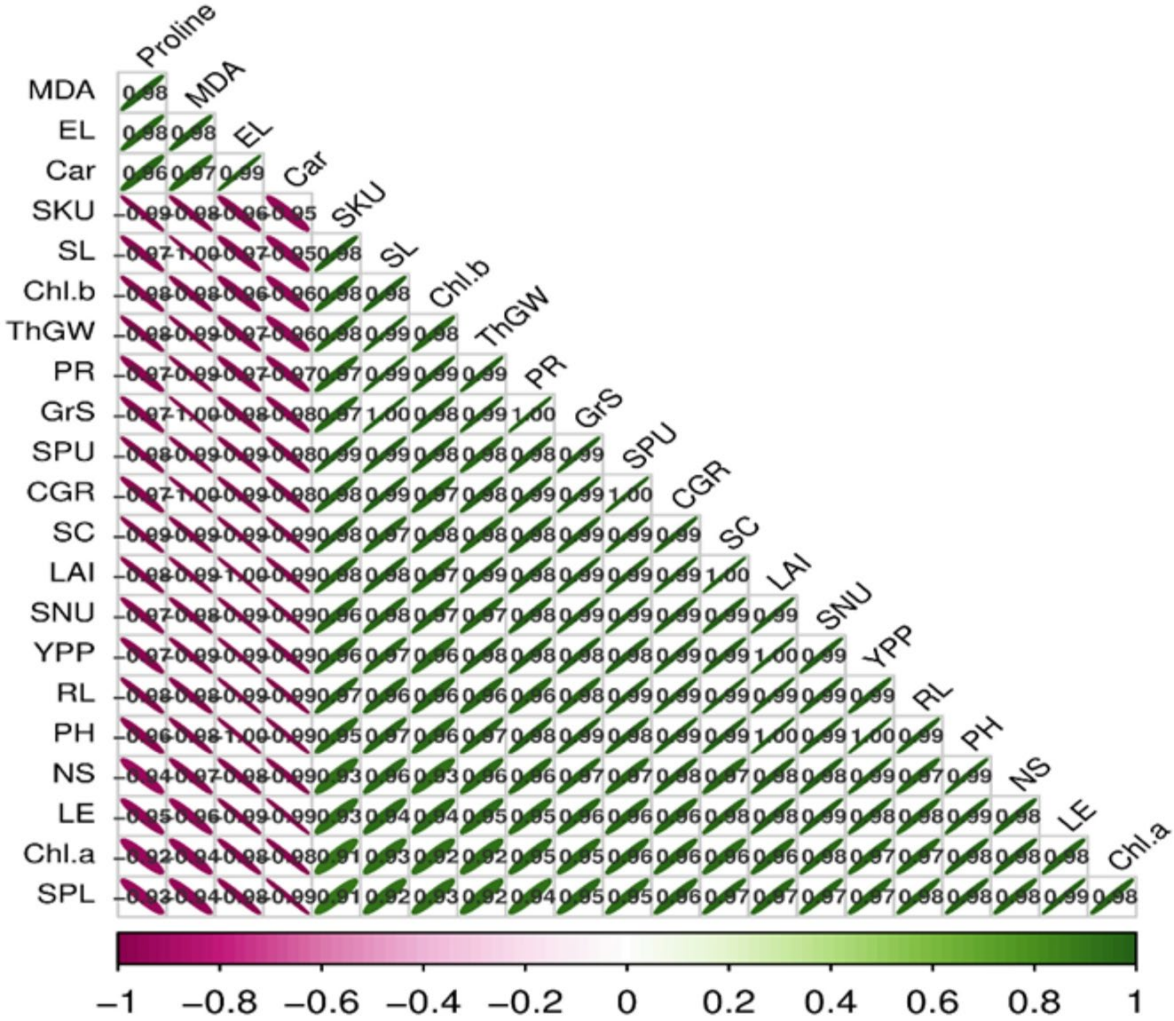
Correlation analysis of the measured parameters of wheat plants under various treatments and drought stress in the field trial experiment. PH = Plant height, SL = Shoot length, RL = Root length, Car = Carotenoids, EL = Electrolyte leakage, SKU = Shoot K uptake, SPU = Shoot P uptake, SNU = Shoot N uptake, Chl a = Chlorophyll a, Chl b = Chlorophyll b, PR = Photosynthetic rate, LAI = Leaf area index, YPP = Yield per plant, CGR = Crop growth rate, SC = Stomatal conductance, LE = Length of ear, NS = No. of spikelets, GrS = Grains per spike, ThGW = Thousand grain weight.

A clear association was evident among all the growth, yield and physiological variables of the wheat plants under drought stress. The attributes, i.e., proline, MDA, electrolyte leakage and carotenoids, exhibited significant negative correlations with all the measured parameters, in contrast the remaining attributes were positively correlated with each other, as shown in Fig. 14.

### Heatmap analysis

**Fig. 15:**
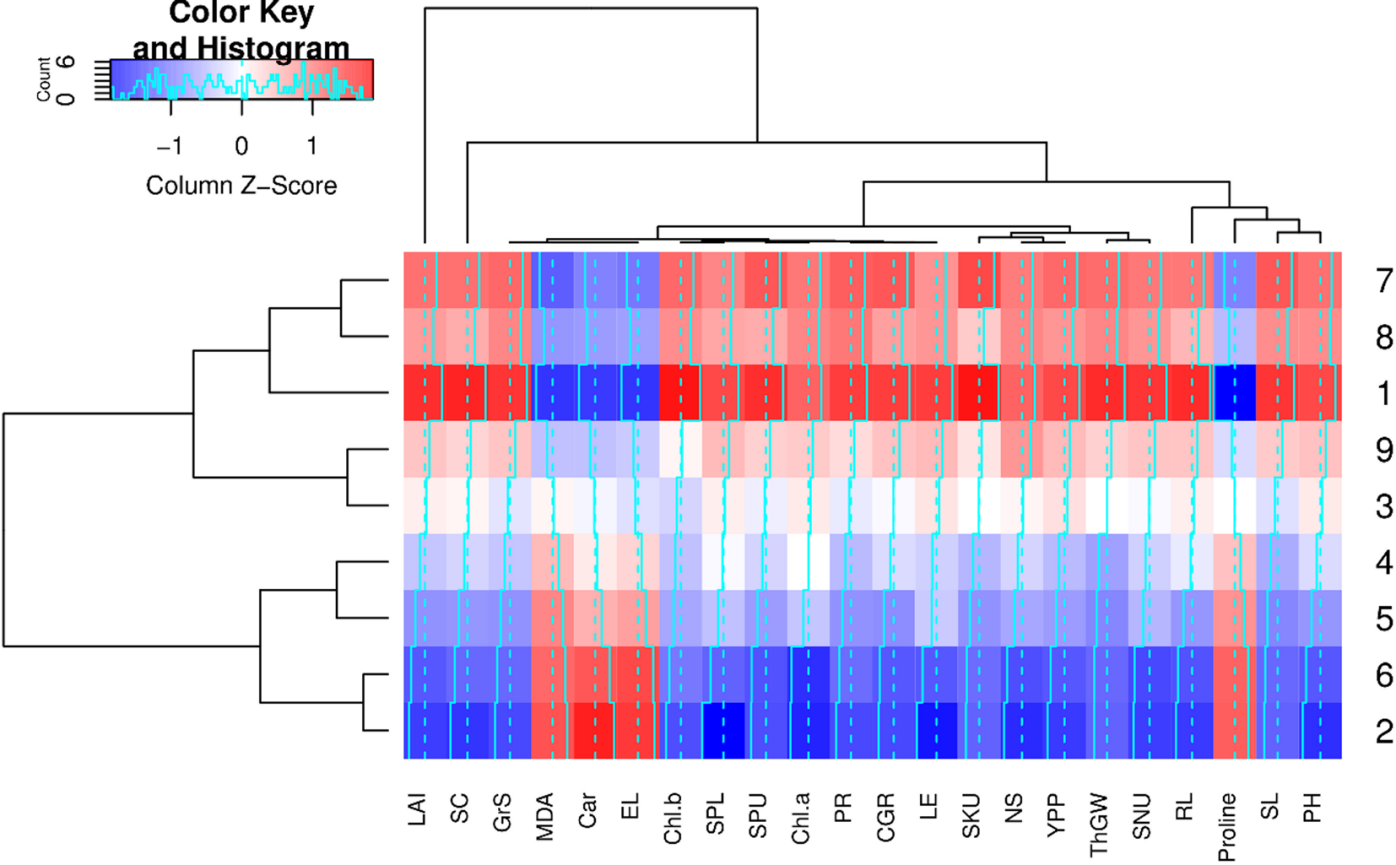
Heatmap analysis of measured parameters of wheat plants under various treatments and drought stress conditions in the field trial experiment. PH = Plant height, SL = Shoot length, RL = Root length, Car = Carotenoids, EL = Electrolyte leakage, SKU = Shoot K uptake, SPU = Shoot P uptake, SNU = Shoot N uptake, Chl a = Chlorophyll a, Chl b = Chlorophyll b, PR = Photosynthetic rate, LAI = Leaf area index, YPP = Yield per plant, CGR = Crop growth rate, SC = Stomatal conductance, LE = Length of ear, NS = No. of spikelets, GrS = Grains per spike, ThGW = Thousand grain weight. (Heatmap was generated using the heatmap package in R (v4.5.1, https://cran.r-project.org/bin/windows/base/).

The cluster heatmap analysis summarized the responses of the plant growth, yield and physiological characteristics of the wheat plants subjected to various treatments to mitigate drought stress (Fig. 7). The heatmap segmented the tested treatments (1–9) into distinct dendrograms within the framework of trait connection. In the columns, 1 to 2 represent the positive and negative controls, respectively, with respect to the applied treatments. On the other hand, treatments 3–9 depict mulching types and microbes used in combination with drought stress. All the characteristics demonstrated differential relationships fluctuating from positive (red) to negative (blue) limits with respect to the treatments used, as shown in Fig. 15.

### Principal component analysis

**Fig. 16:**
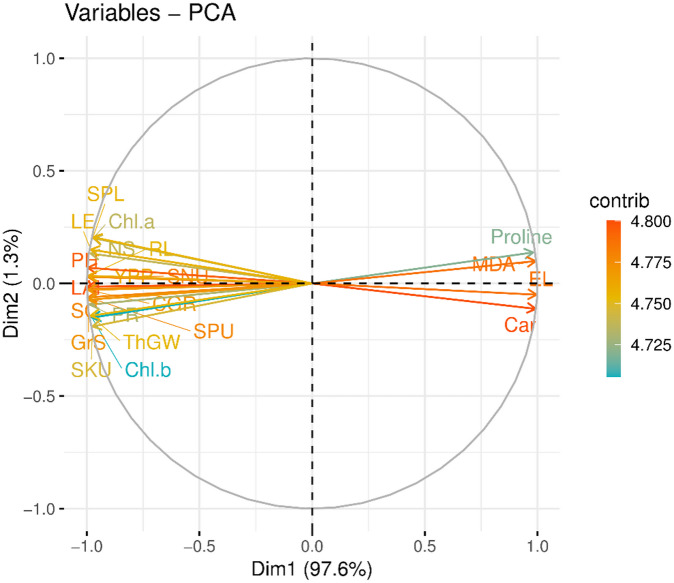
Principal component analysis plot showing loadings of measured parameters and contributions of two principal components (PC1 and PC2). PH = Plant height, SL = Shoot length, RL = Root length, Car = Carotenoids, EL = Electrolyte leakage, SKU = Shoot K uptake, SPU = Shoot P uptake, SNU = Shoot N uptake, Chl a = Chlorophyll a, Chl b = Chlorophyll b, PR = Photosynthetic rate, LAI = Leaf area index, YPP = Yield per plant, CGR = Crop growth rate, SC = Stomatal conductance, LE = Length of ear, NS = No. of spikelets, GrS = Grains per spike, ThGW = Thousand grain weight.

All measured parameters (~ 22 variables) are plotted by employing the statistical tool of principal component analysis (PCA) for dimensionality reduction and a method to simplify a large dataset into clusters while maintaining significant patterns (Fig. 16). The total variability was 98.9%, with a PC1 of 97.6% and a PC2 of 1.3%. Two major groups are observed, with one cluster consisting of EL, Car, MDA, proline and others plotted in the other cluster of the PC1 and PC2 quadrants. The groups (PC1 and PC2) are more or less plotted diametrically opposite to each other in the PC quadrants, with loadings scattered across the plot.

## Discussion

Many studies have provided evidence for improved crop production through the application of plant growth-promoting bacteria under drought stress. Unlike plants, crops do not possess adaptive features to tolerate drought stress^21^. Therefore, it is necessary to find agronomic techniques and tools for sustainable application to increase wheat yield under drought stress conditions^47,48^. The plants without drought stress at the booting stage presented the greatest values for plant height, root length, shoot length, spike length, number of spikelets, leaf area index, 1000-grain weight, grain weight per plant, and crop growth rate^49–51^. The plants subjected to drought stress with no treatment to mitigate drought stress effects presented the lowest plant growth. Similar findings were also reported by previous researchers^52,53^.

For plants subjected to drought stress at the booting stage, the combined application of *A. brasilense* and mulch resulted in higher values for crop growth parameters than did the treatments with only the application of *A. brasilense* or mulch. Previous researchers^38^ reported that plant growth regulator bacteria improve plant growth by alleviating water stress. In another study^52^, this was also reported that inoculation with *A. brasilense* improved the availability of nutrients to plants by providing suitable soil conditions and hence improved plant growth. It also enhances cytokinin production for better cell division and hence improves plant growth^1,54^. *A. brasilense* improves plant growth under drought stress and increases the number of spikelets by providing better organic matter, reducing the amount of chelated compounds and increasing nutrient availability^3,55^. *A. brasilense* also improves plant growth by adjusting plant water characteristics^38^. The number of spikelets increases with increasing spike length^56^. Nutrient availability for plants improves plant growth; hence, the number of spikelets per spike increases through the inoculation of *A. brasilense*^1^. Inoculation with *A. brasilense* improved spike length, and increasing spike length resulted in more spikelets. An increasing number of spikelets results in a greater number of grains and hence a greater grain yield per plant. Similar findings were reported by Zaheer et al.^3^. *A. brasilense* produces many plant growth-related hormones, which result in greater grain weight per plant. Similar findings were reported by Raheem et al.^57^. Iqbal et al.^1^ reported that increased production of cytokinin by *A. brasilense* results in increased 1000-grain weight. Greater CK production also results in better root growth, shoot growth and leaf area^52^. Improved growth parameters resulting from the inoculation of *A. brasilense* have also been reported by Lu et al.^58^. Malondialdehyde (MDA) is a product of lipid peroxidation. The results of the present study revealed an increase in MDA under drought stress with no *A. brasilense* or mulch treatment. Inoculation with *A. brasilense* and the application of mulch to wheat plants reduced the MDA concentration. Similar results were reported by^21^. Electrolyte leakage is used as an indicator of membrane damage under drought stress^59^. Inoculation with *A. brasilense* and mulching reduced electrolyte leakage in wheat plants. Similar results were reported by^60^. The proline content reflects the degree of drought stress in a plant. The results of the present study revealed that plants subjected to drought stress presented the greatest accumulation of proline, whereas plants treated with *A. brasilense* and mulching presented low proline contents. This trend reflected the drought stress level in the wheat plants. Similar results were reported by Zarea et al.^21^, who reported that the accumulation of proline is the result of environmental stress. Biotic stress, such as heavy metals, low temperature, drought and salinity, causes plants to accumulate proline^61^. Under drought stress, plants treated with *A. brasilense* and straw mulch presented greater values of photosynthetic pigments, leaf area, photosynthesis rate and stomatal conductance. In contrast, the plants subjected to drought stress without *A. brasilense* or mulch presented the lowest values. As discussed earlier, the application of *A. brasilense* and mulch improved the availability of nutrients and water to wheat plants. Iqbal et al.^1^ reported that the application of *A. brasilense* enhances cytokinin production to increase cell division, chlorophyll production and leaf area. Similar findings were also reported by many previous researchers^3,52,62^.

Mulching also plays a role in conserving water. Hence, it plays a crucial role in mitigating drought stress. Hu et al.^32^ reported that mulching helps to conserve water in the top 0–80 cm of soil later. In the present study, the availability of water for a longer time resulted in better crop production. Bu et al.^63^ conducted a study in dry lands and reported that direct solar radiation caused more evaporation losses from the soil. On the other hand, mulching can reduce this evaporation. Xiaoli et al.^64^ also reported that mulching increases water availability and hence helps improve wheat production. In this study, wheat straw mulch in combination with *A. brasilense* resulted in better output than did rice straw mulch or plastic mulch alone. Although plastic mulch tends to reduce evaporation losses, better decomposition of straw mulch provides nutrients to wheat plants and results in better crop production. Similar results were reported by Ikram et al.^65^, where wheat straw presented a relatively high decomposition rate, and a relatively high wheat straw decomposition rate provided nutrients to wheat plants and hence resulted in improved crop growth. Similar results were also reported by Sarwar et al.^66^, that water preservation resulted in better wheat production.

The observed improvements in photosynthetic rate, stomatal conductance, and carotenoid contents in wheat plants treated with *A. brasilense* and mulching can be explained by their synergistic effects on plant physiology^3,64^. Inoculation with *A. brasilense* enhances nutrient availability, particularly nitrogen, phosphorus, and potassium, which are essential for chlorophyll synthesis and stomatal regulation^1,5^. Higher nutrient uptake supports greater chlorophyll and carotenoid accumulation, leading to improved light harvesting and photoprotection under drought stress^62,67^. Moreover, *A. brasilense* produces phytohormones such as cytokinins, auxins, and gibberellins, which promote better root architecture and water uptake^68^. This enhanced root system, combined with the moisture conservation capacity of mulches, reduces water stress at the cellular level, maintaining turgor pressure and enabling sustained stomatal opening for efficient gas exchange and photosynthesis^69^. As a result, these treatments significantly improved plant growth parameters, including plant height, leaf area index, and spike development.

At the metabolic level, the reduction in malondialdehyde (MDA) and electrolyte leakage in treated plants reflects lower oxidative stress and better membrane stability^68^. *A. brasilense* inoculation enhances the antioxidant defense system, reducing lipid peroxidation^52^, while organic mulches further alleviate oxidative damage by maintaining favorable soil temperature and moisture^33^. Similarly, the decline in proline accumulation in treated plants indicates a lower drought-induced osmotic imbalance, as water availability and nutrient supply were improved through mulch decomposition and bacterial activity^52,59^. Enhanced nutrient uptake, particularly potassium, plays a vital role in osmotic adjustment and enzyme activation, thereby supporting better grain filling and higher thousand-grain weight^36,70^. Together, these physiological and metabolic adjustments explain why the combined application of *A. brasilense* and wheat straw mulch outperformed other treatments, resulting in improved crop growth rate, spikelet formation, and final yield components^31,35,52,69^. This study has some limitations, as it was conducted at a single site and season, so that results may vary under different climates and soils. Only short-term effects and three mulch types were tested, leaving scope for long-term studies and additional materials. Future work should validate these findings across environments and seasons, explore physiological and molecular mechanisms, and assess economic feasibility to support broader adoption in sustainable agriculture.

## Conclusion

The ability of drought stress to mitigate food for increasing populations is becoming a severe issue. To ensure food security, sustainable solutions for crops are needed. *A. brasilense* is beneficial for crop growth under drought stress. Mulching also provides a sustainable solution to mitigate this situation. Plastic mulch is efficient at conserving water, but straw mulch has additional benefits in terms of nutrient provision through in-field decomposition. In this study, drought stress was applied to wheat plants at the booting stage, and the effects of wheat seed inoculation with *A. brasilense* and mulching were evaluated. The results revealed that the combined application of *A. brasilense* with mulching had better effects on plant growth parameters, including plant height, root length, shoot length, spike length, number of spikelets, leaf area index, 1000-grain weight, grain weight per plant, crop growth rate, chlorophyll content, stomatal conductance, and antioxidants. Straw mulch resulted in better results than plastic mulch. Additionally, wheat straw mulch had a greater effect on wheat plants than did rice straw mulch. These findings highlight the potential of combining *A. brasilense* inoculation with straw mulching as a cost-effective and eco-friendly practice for sustainable wheat production under drought conditions. Such approaches could be integrated into climate-smart agricultural policies to improve crop resilience and ensure food security.

## Data Availability

Data will be made available upon reasonable request from the corresponding author, Kamran Ikram.
